# Unmasking DNA Resonances by Suppression of Hyperpolarized Water

**DOI:** 10.1021/jacs.6c07731

**Published:** 2026-07-24

**Authors:** Milan Zachrdla, Ertan Turhan, Michala Bučková, Lukáš Trantírek, Dennis Kurzbach

**Affiliations:** † Institute of Biological Chemistry, Faculty of Chemistry, 27258 University of Vienna, Währinger Str. 38, 1090 Vienna, Austria; ‡ Central European Institute of Technology, 37748 Masaryk University, 625 00, Brno, Czech Republic; § National Centre for Biomolecular Research, Faculty of Science, 37748 Masaryk University, Kamenice 5, 625 00 Brno, Czech Republic

## Abstract

Buffers based on hyperpolarized water (HyperW) generated by dissolution dynamic nuclear polarization (dDNP) enable orders-of-magnitude signal enhancements in biomolecular NMR and residue-resolved access to a range of target systems at near-physiological concentrations and conditions. At the same time, the benefits of this signal enhancement are fundamentally counteracted by an overwhelming water signal that obscures most of the ^1^H spectrum. Therefore, nonisotopically enriched targets, including most nucleic acids, the second-most-abundant class of biomolecules, remain largely inaccessible to dDNP applications. Here, we introduce a versatile postprocessing strategy based on singular value decomposition that selectively removes the obscuring HyperW contribution while preserving full biomolecular hyperpolarization. This approach eliminates this major downside of biomolecular dDNP experiments in aqueous environments and restores access to the largest share of the ^1^H spectral range. Our HyperW signal suppression method enabled us to access previously masked hyperpolarization reservoirs across a range of DNA targets. (i) We were able to monitor multiple site-resolved polarization transfers from HyperW to DNA via exchange-relayed NOE pathways in real time, in a single experiment, for all DNA moieties (aromatic, amino, carbohydrate) distributed across the full range of the ^1^H spectrum. In contrast, hyperpolarized NMR was previously largely limited to imino resonances. (ii) Application to noncanonical, structurally distinct i-motif and G-quadruplex DNAs allowed us to selectively hyperpolarize distinct molecular regions, providing real-time insight into solvent interactions that are invisible to conventional NMR. Finally, the method is broadly applicable, as shown with five diverse target molecules, and thus provides a versatile route to biomolecular ^1^H-detected dDNP in aqueous environments.

## Graphical abstract

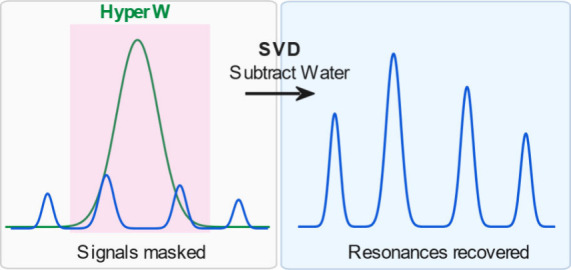

## Introduction

Nuclear magnetic resonance (NMR) spectroscopy remains the only technique capable of resolving residue-specific information in biomolecular systems in their native solution environment. This capability has made NMR indispensable for studying the structural dynamics of small- to medium-sized nucleic acids and proteins. However, its applications are fundamentally constrained by its low intrinsic sensitivity, which limits investigations to relatively high concentrations and precludes access to many transient or low-populated states that provide only little NMR signal strength.^1,2^

As a result, considerable effort has been devoted to overcoming this sensitivity bottleneck, including the development of higher magnetic field strengths >1 GHz,^3,4^ advanced probes and pulse sequences,^5–7^ and, more recently, machine-learning–assisted reconstruction strategies.^8,9^ Among the sensitivity-enhancement approaches, hyperpolarization techniques, in which the nuclear spin system is driven far away from equilibrium, have emerged as a particularly powerful strategy, enabling signal enhancements by several orders of magnitude.^2,10^ Dissolution dynamic nuclear polarization (dDNP) stands out in this context due to its exceptional sensitivity gains (up to 1,000-fold for ^1^H and 50,000-fold for ^13^C) and broad applicability across chemical and biological systems.^11–13^

However, despite its promise, dDNP faces a critical limitation for biomolecular applications. In a dDNP experiment, the sample is pretreated ex-situ by microwave irradiation at cryogenic temperatures (*T*_DNP_ ∼ 1 K) before rapid melting and transfer to an NMR spectrometer for detection. The requirement to shuttle samples from cryogenic hyperpolarization conditions to ambient NMR detection imposes harsh physicochemical constraints. Indeed, direct use with biological systems that rely on noncovalent secondary and tertiary structures is mostly impossible. These targets are typically denatured in dDNP experiments.^14^ To circumvent this, hyperpolarized water (HyperW) has been recently introduced as a polarization relay medium.^14–17^ Only the buffer becomes hyperpolarized ex-situ and is then mixed in situ, within the spectrometer, with a target molecule under mild conditions. Transfer of signal intensity to the target biomolecules then takes place via proton exchange and NOE-mediated pathways.^18^ HyperW has already enabled a multitude of applications at near physiological conditions and, importantly, also concentrations^19^ – from real-time monitoring of protein interactions^17,20–22^ to detection of invisible states in nucleic acid folding.^23,24^ However, one problem still remains to be overcome when aiming to capitalize on the full potential of hyperpolarized water: the overwhelming water signal and associated baseline distortions obscure large portions of the proton spectrum, effectively limiting analysis to resonances well separated from water. In other words, only signals above ∼ 8 ppm or below ∼ 2 ppm are amenable to investigation. The situation is a true dilemma, the stronger the water hyperpolarization, the broader the water resonance and, thus, the smaller the analyzable fraction of the spectrum.

Figure 1 demonstrates this problem using a deoxyribonucleic acid (DNA) as an example, the most recent class of biomolecular target molecule added to the dDNP portfolio.^23^ Proton exchange of imino and amino residues with HyperW introduces hyperpolarization to the target, then nuclear Overhauser effects (NOEs) transfer the polarization from the labile protons to the rest of the DNA (Figure 1a). However, only imino and amino resonances at >8 ppm could so far be read out.^23^ Figure 1b-1c shows that, with a typical water signal enhancement of ca. 200-fold, the aliphatic DNA signals (c­(DNA) = 125 μM) are barely visible next to the overwhelming solvent line. They are deeply buried in baseline distortions and cannot be analyzed without prohibitively large errors. Unfortunately, established solvent-suppression methods^25^ fail in the context of HyperW. Pulse sequence editing cannot be used, as it would simultaneously attenuate the very hyperpolarized reservoir needed for analyte enhancement. Differential spectroscopy, frequently applied in in-cell NMR studies,^26^ is ineffective in this case because variations in the HyperW line shape caused by radiation damping and nonlinear, partially chaotic^27^ behavior prevent reliable subtraction of the water signal. Likewise, model-based fitting and subtraction are not viable, as the HyperW signal exhibits pronounced asymmetry and nonideal behavior that cannot be robustly parametrized.^28^ Selective excitation of only nonwater protons, of course, remains an option, but large parts of the spectrum covered by the water resonance simply remain undetectable.

**Figure 1:**
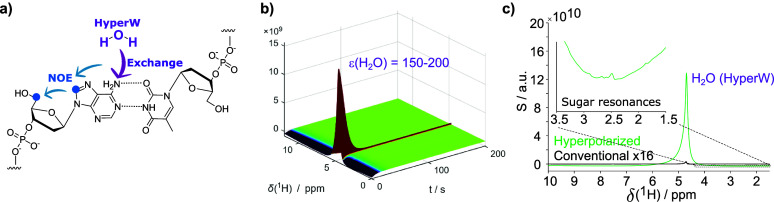
Hyperpolarized water boosts signals, but also obscures NMR spectra of biomolecular DNA targets. **a)** Hyperpolarized water (HyperW) exchanges polarization with DNA via exchange with labile imino and amino protons involved in hydrogen-bonded base pairs. The hyperpolarization is subsequently relayed via NOE to nearby nonexchangeable aromatic- and sugar-hydrogens (arrows guide the eye). The NOE transfer pathways are specific to the structural context and may concurrently operate at the intranucleotide, intrastrand, or cross-strand level across all protons. **b)** Typical HyperW decay (ω_L_ = 500 MHz, *T* = 25 °C) after injection into an NMR spectrometer. The water hyperpolarization has a lifetime of ca. 1 min. The signal enhancement ε can reach up to 200-fold, relative to the signal intensity of the final dilute sample. In the case of pure HyperW, enhancements reached 420-fold under our experimental conditions (see Materials and methods for details). The major problem with HyperW is the strong water resonance during the early stages of the detection period. It masks large parts of the ^1^H spectrum and distorts the baseline, thus rendering most resonances unusable. **c)** A DNA dissolved in an aqueous buffer. Green: with hyperpolarized water; black: with conventional water. The typical concentration of a biomolecular target is much lower than that of the water. The inset shows that, as a result, the DNA sugar resonances are buried within the baseline distortion caused by the HyperW.

Here, we show that overcoming this limitation is possible with a versatile postprocessing strategy that selectively suppresses hyperpolarized water contributions and associated spectral distortions while preserving hyperpolarized biomolecular signals. This approach enables access to (almost) the full ^1^H spectrum in hyperpolarized aqueous NMR experiments, thus rendering a large pool of previously out-of-reach hyperpolarized signals now accessible.

We demonstrate the potential of this method using five molecular systems, including two distinct intramolecular tetraplex-based DNA motifs (Supporting Information Table S1), namely the i-motif (iM)^29^ and parallel G-quadruplex (GQ) DNA.^30^ These tetraplex systems were selected based on (i) biological relevance - both iM and GQ DNA are among the most extensively studied noncanonical DNA structures due to their emerging roles in replication and gene regulation;^31,32^ (ii) their aromatic and sugar proton chemical shifts are representative of those observed across a wide range of DNA and RNA motifs, with many resonances located close to the water signal; and (iii) although both iM and GQ contain well-defined, structurally homogeneous cores, their fundamentally different architectures give rise to distinct NOE networks for exchange-NOE-mediated polarization transfer, making them suitable systems for method assessment.

The observation of hyperpolarized sugar and aromatic resonances in these systems highlights the capability of our approach to access previously obscured regions of the ^1^H spectrum. Importantly, we show that access to these resonances is more than a technical advance. By resolving aromatic GQ signals with substantially higher sensitivity, we were able to monitor, in real time, the hyperpolarization flow from water to individual GQ moieties across several resonances in parallel. Thus, we could selectively boost and track loop and tail signals from the bulk. Thus, a wealth of formerly masked kinetic traces becomes immediately accessible.

Beyond this specific application, the presented method is demonstrated to be broadly applicable and provides a route toward ^1^H-detected biomolecular dDNP in aqueous environments, including noncovalently folded targets. By removing the dominant spectral barrier imposed by water, it opens the way to broaden hyperpolarized NMR from a niche technique into a versatile tool, no longer limited to downfield-shifted imino and amino resonances.

## Results and Discussion

Our approach, inspired by the early work by Won and co-workers, is visualized in Figure 2.^33^ It is based on the decomposition of the NMR time-domain signal, the free-induction decay (FID). First, the FID is transformed into a Hankel matrix, followed by a singular value decomposition (SVD); an approach typical, e.g., in Cadzow-style denoising.^34–36^ Second, and this is where our approach deviates from existing strategies and becomes tailored for hyperpolarization, we capitalize on the intrinsic structure of HyperW data: a dominant, highly correlated water resonance coexisting with comparatively weak analyte signals. As a result, the singular values (σ_i_) associated with HyperW are orders of magnitude larger than those of the analyte, effectively clustering as low-rank singular values. These reflect a strongly correlated temporal evolution of the HyperW resonance, in contrast to the multifrequency, weakly correlated analyte contributions. This separation enables a clear discrimination between solvent and biomolecular contributions. Third, we reconstruct the HyperW signal from the dominant singular values (cyan spectrum) and subtract it from the full reconstruction (black), yielding the pure hyperpolarized analyte spectrum (red) with minimal residual water contributions. Full details are provided in the experimental section.

**Figure 2:**
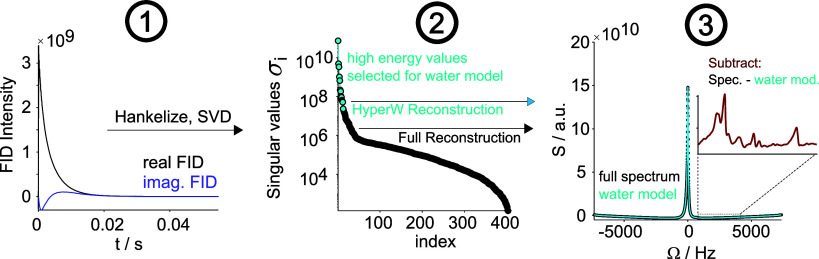
Workflow of the HyperW-suppression procedure, which selectively eliminates hyperpolarized spectral solvent components while preserving the target hyperpolarization. **1.** The hyperpolarized FID (HyperW + target molecule) is transformed into a Hankel matrix followed by SVD as typical in Cadzow-type denoising procedures. **2.** The resulting singular values are ordered by intensity. The most intense ones (cyan) reflect the dominant water contribution (note the logarithmic scale). **3.** One can now reconstruct the HyperW contribution from the cyan intense singular values (reverse SVD, followed by re-Hankelization) and subtract the resulting water model from the full spectrum. The resulting differential spectrum (red) retains only the spectral contributions of the dissolved biomolecule (red inset), which were previously buried beneath the overwhelmingly strong HyperW resonance. The details of this procedure are described in the Materials and Methods section and the Supporting Information.

With our approach, we were able to combine SVD-based^33,35^ approaches to postprocessing with hyperpolarized NMR and, in particular, with HyperW experiments, and as such render observations possible and resonances detectable that would otherwise remain out of the scope of existing approaches.

First, we demonstrate the capacity of this approach using an iM DNA construct (Supporting Information – Table S1, referred to, in line with previously published studies, as T121-6).^29^ In our experiments, HyperW was produced by DNP at *T*_DNP_ = 1.4 K and *B*_0,DNP_ = 6.7 T using 15 mM TEMPOL radicals as polarization agents dissolved in 70/30% (v/v) H_2_O and glycerol-d_8_ (used as a cryoprotectant). The hyperpolarized sample was dissolved with a burst of hot D_2_O (ca. 250 °C under 15 bar overpressure) and then transferred to a 500 MHz NMR spectrometer, where it was mixed with a solution of the target DNA using a home-built device (HySSS.v2).^37^ After completion of the mixing procedure, NMR acquisition started with a train of 1° θ-pulses applied every second. Note that the target DNA was prepared in a deuterated buffer.

The raw-detected first spectrum directly after mixing of the T121-6 iM with HyperW (Figure 3a), shows only a distorted, very broad hyperpolarized water signal at first glance. Figure 3b shows the associated molecular structures. Subtracting the water resonance using our procedure then reveals the underlying ^1^H spectrum of the DNA component (Figure 3c, blue). Only minor residual water and glycerol signals remain; clearly discernible from real signals as sharp baseline oscillations at 4.7 and 3.5 ppm, respectively. The distortions associated with these oscillations directly interfere with the expected positions of H3′ protons (typically 4.4–5.0 ppm; Figure 3b) and preclude observation of sugar H1′ and aromatic H5 resonances, which typically appear in the 5–6 ppm region. However, apart from these remainders, the spectrum looks as expected compared to a conventionally recorded NMR spectrum of the T121-6 DNA (Figure 3c red): All resonances are at the expected positions, with line widths matching those observed in the reference experiment. Only the signal amplitudes differ, again as expected, since the transfer of the solvent hyperpolarization to the different sites within the DNA proceeds with varying efficiency, so that different moieties are enhanced to varying degrees. The inset in Figure 3c (right-hand side) shows the heterogeneity of the signal enhancement ε, with values spanning 3 to 200 across the different resonances. (Note that preservation of analyte intensities depends on selecting a truncation rank within a defined window, Figure S1–S2, where solvent contributions are captured while analyte components remain unaffected). The enhancements were calculated via the signal intensity ratio between the hyperpolarized and reference spectra after decay of the hyperpolarization of the exact same sample (all details in the Materials and Methods Section).

**Figure 3:**
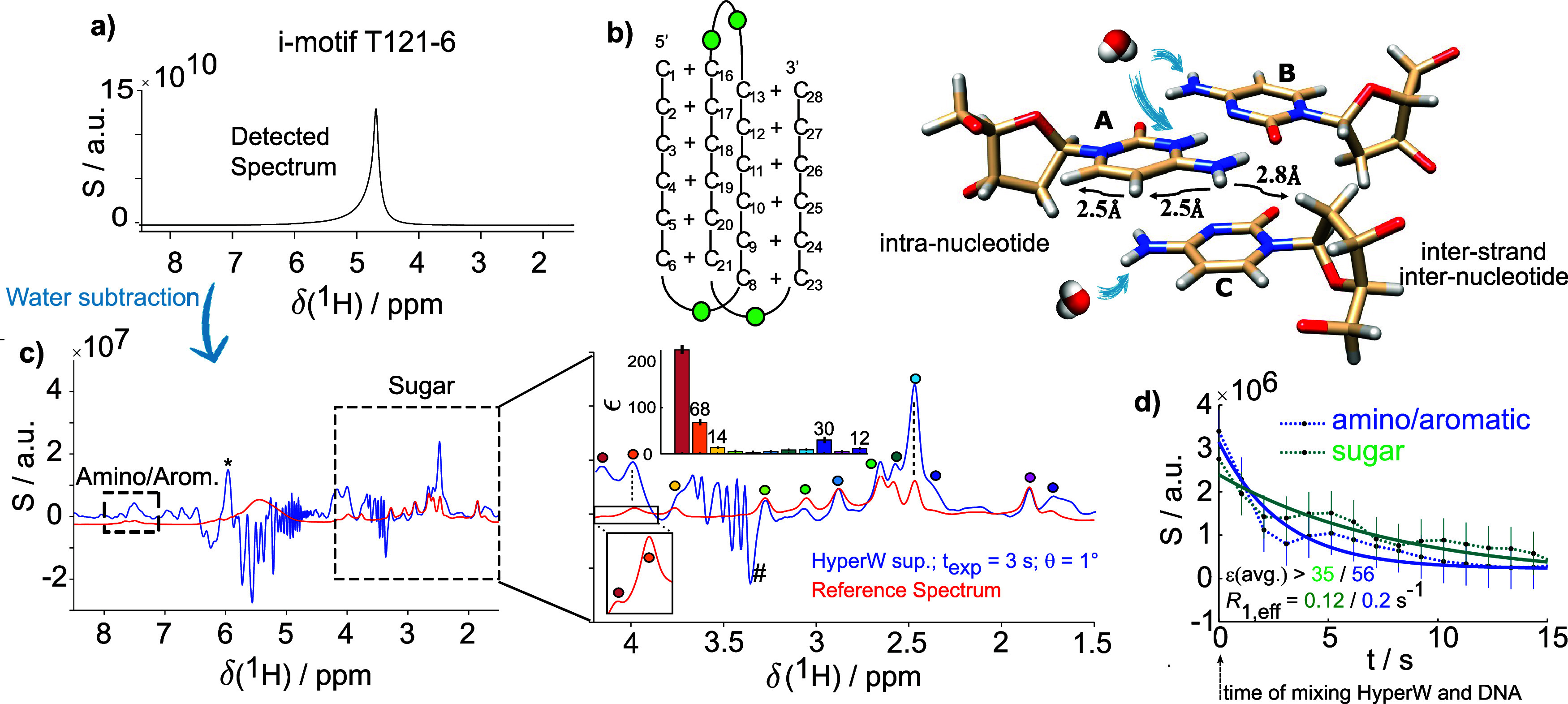
Suppression of the HyperW component allows real-time monitoring of the ^1^H spectrum of an i-motif (T121-6) DNA. **a)** The raw hyperpolarized ^1^H spectrum of T121-6 in HyperW. Note how the DNA signal is entirely lost in the overwhelming water signal. **b)** The structure of the DNA motif is indicated. Right: Section of i-motif stem structure (PDB ID: 1ELM) showing the most efficient NOE-mediated polarization transfer pathway starting from HyperW to the exchangeable amino protons (cytosine A) in the solvent-accessible groove to the aromatic H5/H6 within the same residue and to H2′ of the sugar moiety (cytosine C). Note that cytosines A, B, and C are located in different strands. **c)** Subtraction of the HyperW component reveals the underlying hyperpolarized T121-6 spectrum (blue; θ = 1°; ns = 3). The comparison with a conventionally measured reference (red; θ = 90°; ns = 128) demonstrates that our method faithfully reproduces the expected resonances. Note that not all peaks are hyperpolarized to the same extent. Only a subset receives efficient NOE from the proximate imino protons. The inset on the right-hand side shows the heterogeneous signal enhancements ε for the different resonances of the sugar region (maximum ε: > 200-fold; average ε: 35; error bars are indicated). Selected enhancements are numerically indicated. The # indicates residual signals from glycerol-d_8_. Dashed lines demonstrate the correspondence of resonance positions. * indicates residual water signal. The inset at the bottom left zooms in on the weak reference signal at 4.2 ppm. **d)** Signal intensity decay of the aromatic/amino and the sugar bulk resonances indicated in panel b. After mixing of T121-6 with HyperW, the hyperpolarization transferred to the aromatic protons decayed with a rate constant of 0.2 s^–1^, and the sugar with 0.12 s^–1^. The average signal enhancement was >56 and >35, respectively. These data show that our approach efficiently removes the HyperW line while preserving the target hyperpolarization.

Note that the high degree of solvent deuteration upon detection (nominally 98.5%) causes imino and amino protons to become partially deuterated, which artificially attenuates signals in the absence of hyperpolarization and relative to a standard NMR buffer containing only 10% D_2_O. This effect was regarded in detail in reference.^23^ For the aliphatic protons reported in Figure 3c, proton exchange on the time scale of the dDNP experiment can be neglected, such that the reported enhancements are representative of the actual signal gain. However, amino resonance and aromatic protons might overlap in the region between ∼7 and 8 ppm, potentially influencing the bulk enhancement reported in Figure 3d.

At the same time, the degree of imino protonation also determines the starting point for the exchange-relay NOE. Fewer hyperpolarized labile sites, evidently, lead to less starting polarization to be relayed to the nonexchanging sites.^23^

Because detection of the HyperW-boosted spectrum takes only a second and the 1° excitation pulse reads only a small fraction of the available polarization, it is straightforward to obtain time-resolved data on individual resonances. Figure 3d shows the intensity of the bulk integral over the aromatic/amino signals as well as over the sugar signals as a function of time after mixing (corresponding to *t* = 0 in the figure) HyperW and T121-6. As HyperW continuously replenishes the magnetization on the target DNA, the intensity decays at slow *R*_1,eff_ rate constants of ∼0.2 s^–1^ and ∼0.12 s^–1^, respectively. Using the signal strength at *t* = 0, an average ε of >56 and >35 can be estimated, respectively. (For some residues, the thermal equilibrium reference remained below the detection threshold; thus, the inequality sign.)

These observations can be rationalized by the structural features of the i-motif. In T121-6, as in other i-motifs, the spatial arrangement – comprising two intercalated parallel duplexes stabilized by hemiprotonated C·C^+^ base pairs – places exchangeable amino protons in close proximity to both aromatic (H5) and sugar (H2′) protons, with comparable distances (∼2.48 and ∼2.75 Å), enabling efficient direct polarization transfer to both moieties. However, slightly shorter distances and more favorable geometry for amino–aromatic contacts are expected to promote more efficient polarization transfer to aromatic protons, consistent with their higher observed enhancement (Figure 3c-d). Notably, resonances in the aromatic/amino region exhibit extensive spectral overlap due to the limited chemical-shift dispersion characteristic of i-motif DNA with long C-tracts. Consequently, only a composite signal contribution can be analyzed, precluding residue-specific decomposition analogous to that performed for the aliphatic region.

In contrast, polarization transfer within the sugar moiety can be further amplified by efficient relay pathways, supported by shorter intrasugar distances (H2′–H2″, 1.79 Å; H2′–H3′, 2.25 Å) compared to the corresponding aromatic contacts (H5–H6, 2.45 Å). This network of short-range couplings may facilitate redistribution and retention of polarization within the sugar spin system, contributing to its slower apparent decay. Thus, the structural constraints are consistent with preferential polarization transfer to aromatic/amino sites followed by partial relay and longer-lived polarization within the sugar moiety.

Altogether, the T121-6 data demonstrate that our approach enables detection of low-abundance species dissolved in HyperW (c­(DNA) = 0.125 mM vs 870 mM H_2_O, corresponding to 0.15% (v/v) HyperW) at significantly enhanced signal intensities, without adversely affecting resonance positions or line widths. At the same time, our data demonstrate that time-resolved tracking of polarization flow across individual resonances of the DNA becomes possible. Thus, access is provided to coupled solvent-exchange/NOE pathways and their underlying structural determinants.

In the next step, this capacity is put to use to explore the conformational space of a c-Myc-derived GQ DNA. In contrast to the T121–6 iM, in this DNA structure, comprising stacked guanine tetrads formed by Hoogsteen hydrogen bonding and stabilized by monovalent cations, the spatial arrangement of guanine tetrads and connecting loops leads to a markedly different distribution of interproton distances. Concerning the distances within the stacked G-tetrads, the exchangeable amino protons (N2–H_2_) are positioned closer to aromatic (H8) protons (∼ 3.2 Å) than to sugar protons, while contacts to the sugar moiety are generally longer and more variable (>4.1 Å). This geometry is expected to promote more efficient polarization transfer to aromatic protons, while transfer to the sugar moiety is significantly less favored, even via relayed pathways through aromatic sites. Thus, the c-Myc GQ provides a counterpoint to the T121-6 iM, allowing us to stress-test our approach.

Figure 4a shows the structure of the c-Myc GQ and Figure 4b shows the HyperW-suppressed spectrum. As in the case of T121-6, the spectrum quantitatively reproduces resonance positions and line widths, particularly evident in the sugar resonances between 1.5 and 3.5 ppm, underscoring the robustness of our HyperW suppression approach. One exception is a strong signal at ∼2 ppm that is very weak in the HyperW spectrum (see inset in Figure 4b). This composite resonance arises from thymine methyl groups (residues 7, 11, 16) located in disordered loops and not involved in base pairing.^38^ Their exchangeable imino and amino protons undergo rapid exchange with water, resulting in severe broadening beyond detection. Very likely, these residues do not receive observable hyperpolarization, as efficient polarization transfer through NOEs requires slowed proton exchange, typically achieved through hydrogen bonding.

**Figure 4:**
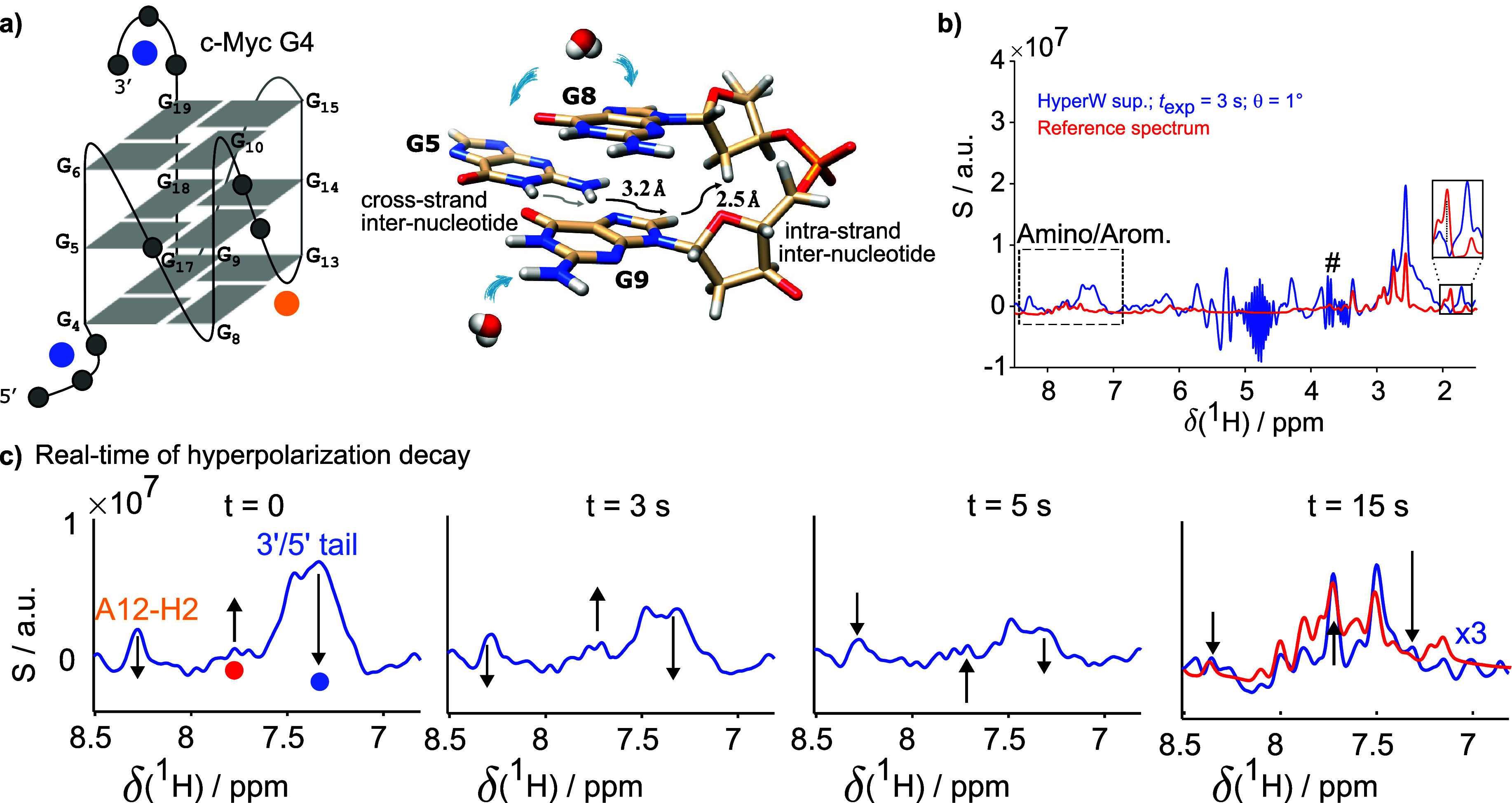
HyperW suppression enables real-time monitoring of hyperpolarization in G4 quadruplexes. **a)** Structure of the c-Myc G-quadruplex, the 5′/3′ tails and the internal loop region containing residue A12-H2 are indicated by blue and yellow dots, respectively. Right: Section of the c-myc GQ structure showing the most efficient NOE-mediated polarization transfer pathway starting from HyperW to the exchangeable amino protons of the G5 in the solvent-accessible groove to the H8 proton of the Hoogsteen base-paired G9 within the same G-tetrad, and subsequently to the H2″ proton of the n–1 guanosine (G8) in the stacked tetrad. **b)** HyperW-suppressed spectrum of c-Myc G4 (blue; θ = 1°; ns = 3), in comparison to a conventional reference (red; θ = 90°; ns = 128). Again, the ribose resonances were correctly reproduced in terms of resonance frequencies. As expected, signal intensities reflect the nonequilibrium nature of the dDNP experiment. # points toward glycerol-d_8_. **c)** Zoom on the aromatic region highlighted in panel (b), at different time points after mixing HyperW with G4 (time on top of the panels). The arrows indicate resonances that are strongly hyperpolarized and then decay within ca. 15 s (yellow, blue; assigned to A12-H2 and the 5′/3′ tail regions) or that slowly grow/remain constant (red; representing the bulk equilibrium spectrum). After 15 s, the reference and the dDNP sample display the same spectrum. These data show that the loop/tail residues of the GQ are preferentially hyperpolarized in HyperW.

Note that the above-mentioned pathways that transfer water hyperpolarization to T121-6 and c-Myc GQ DNA are also supported by ^1^H–^1^H NOESY (Supporting Information Figure S3).

However, in contrast to the HyperW-suppressed spectrum of T121-6, the aromatic/amino region exhibited a distinct behavior, as revealed by the time-resolved spectra (Figure 4c). A resonance at 8.4 ppm and a set of overlapping signals between 7.1 and 7.5 ppm were strongly hyperpolarized and decayed to naught within 15 s. The former was assigned previously to the A12-H2 proton in the internal propeller loop GQ region (Figure 4a).^30^ The latter region comprises exclusively resonances from T1-H6, and A21-H2/H8 and A22-H2/H8, all residues involved in the formation of dynamic capping structures at the 5′ and 3′ termini^28^ (assignment in Figure S4 of the Supporting Information). Thus, although it is impossible to distinguish individual signals due to crowding, a coarse assignment of the ∼7.1–7.5 ppm bulk signal to the capping structures at GQ 5′/3′ tails can be made.

Next, we extracted the signal intensity time traces of the three GQ resonances marked in Figure 4c (A12-H2, 5′/3′ tail, and the bulk signal) and plotted them as a function of time together with the HyperW intensity (Figure 5a). The data were then fitted to a coupled kinetics model including the hyperpolarization decay, the intrinsic nuclear signal, and the relay of hyperpolarization to the three different sites (all details in the experimental section). This yielded a water proton polarization enhancement of approximately 202-fold, confirming efficient initial hyperpolarization, and the HyperW polarization remained alive for ca. 30 s. A fraction of this polarization is transiently relayed to the 5′/3′ tail residues as well as to A12-H2, with transfer rates σ fitted to 0.04 and 0.01 s^–1^, respectively. Notably, the A12-H2 trace deviates from monoexponential decay, consistent with the presence of a NOE-relayed polarization build-up before decaying with an effective rate constant of *R*_2_ ≈ 0.39 s^–1^.

**Figure 5:**
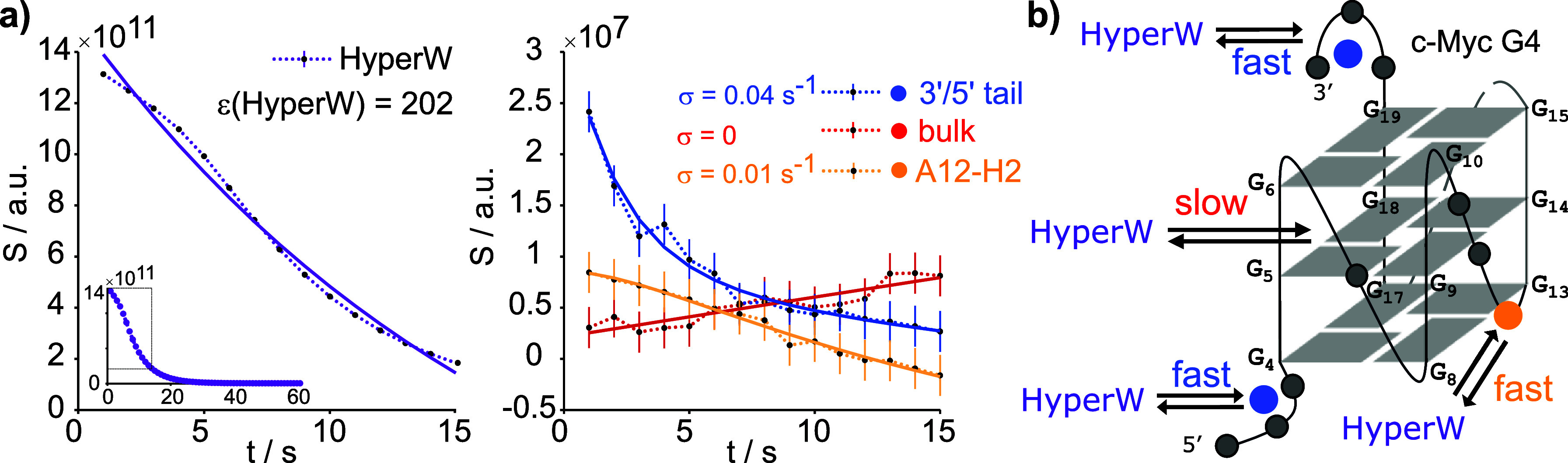
Real-time hyperpolarization monitoring and suggested HyperW-GQ transfer mechanism. **a)** Left: Decay of the HyperW relaxation with time (dots: data, purples line: fit). The overall signal enhancement was 202. Note how the initial data looks nonexponential due to radiation damping. The inset shows the full-time trace. Error bars are within the data points. The water polarization was alive for ca. 30 s. Right: Intensity time traces (black dots) of the equilibrium-state signal and fit (solid lines) and of the A12-H2 and 5′/3′ tails. The transfer rates from HyperW were 0.01 and 0.04 s^–1^, respectively. The residual bulk signals (red) are present with low intensity throughout the entire detection period and slightly recover intensity after mixing with HyperW, likely due to altered relaxation pathways. **b)** Phenomenological model consistent with the observed kinetics. HyperW transfers polarization to the loop regions with high efficiency. However, the folded core of the GQ does not receive any hyperpolarization, allowing us to selectively probe the loop residues.

In contrast, the bulk signal displayed a slow, gradual buildup to a finite plateau, leading to an effective transfer rate σ fitted to 0. This behavior is most plausibly attributed to changes in relaxation properties upon mixing with hyperpolarized water, leading to an apparent recovery of signal intensity. While exchange-mediated transfer from the loops could, in principle, contribute to this buildup, the kinetic fit does not support a measurable exchange pathway.

We want to stress that this observation of site- and time-resolved kinetics would be impossible without hyperpolarization, as the intramolecular NOE pathways would be too weak for real-time NMR readout at Boltzmann polarization in thermal equilibrium. At the same time, all earlier implementations of the HyperW technique would have masked the underlying resonances. In contrast, our approach straightforwardly reveals time-resolved data at high sensitivity that allows us to discriminate exposed loop regions in the GQ from the bulk resonances through selective hyperpolarization (Figure 5b). Similar approaches in the context of intrinsically disordered proteins^21,39–41^ have been reported in the past and are here expanded to DNA, enabling site-selective spectroscopy beyond the typical imino/amino correlation spectra.

Three more examples of the broad applicability of our approach are provided in the Supporting Information (Figures S5–S6).

Finally, it should be noted that machine learning (ML) strategies for handling solvent signals are also currently under development and might provide an alternative to the presented method.^42,43^ However, the extremely dominant HyperW resonance produces a very clear low-rank structure in the singular value spectrum, enabling direct and physically transparent separation between solvent and analyte contributions. Thus, unlike many ML approaches, the present strategy does not require training data sets or black-box optimization procedures.

In this context, it should be noted that conventional SVD-based approaches did not become widely adopted for standard NMR solvent suppression precisely not least because ordinary solvent signals often do not dominate sufficiently for clean singular value separation. HyperW data fundamentally change this situation because the hyperpolarized-solvent signal exceeds the analyte signal by orders of magnitude (cf. Figure S7).

Further, note that our approach leaves residual oscillatory traces of the suppressed water and glycerol signals, thus still obscuring small fractions of the spectrum. Complete suppression cannot be achieved with SVD, as the weak analyte signals are dampened before full suppression is achieved. Potentially, ML-based approaches might, thus, be superior in this regard.

## Conclusions

We introduce a strategy that overcomes a key bottleneck of hyperpolarized aqueous NMR by selectively suppressing the overwhelming water signal while preserving biomolecular target hyperpolarization. This enables, for the first time, the comprehensive recovery of ^1^H spectra and associated kinetic information from spectral regions previously obscured by HyperW, thereby rendering almost the full spectral width accessible under dDNP conditions.

Importantly, the approach allows for the extraction of multiple time-resolved signal traces in parallel from a single experiment, providing direct access to hyperpolarization kinetics without the need for repeated measurements. At the same time, these traces are effectively read out via site-specific detection, as signals from exposed residues are more efficiently hyperpolarized. Thus, rather than merely enhancing signal intensity, the method enables real-time mapping of polarization flow through specific biomolecular moieties.

The broad applicability of the method is evidenced by its successful application to five distinct systems, highlighting its general utility for hyperpolarized ^1^H NMR in aqueous environments, enabling access to biomolecular substrates under near-physiological conditions and approaching concentrations close to those of cell-free DNA.^44^

Taken together, the presented method provides an avenue for hyperpolarized ^1^H NMR in aqueous environments to develop from a scope-restricted technique into a broadly applicable means for studying biomolecular structure and dynamics. By removing the dominant spectral contribution imposed by HyperW, it opens new avenues for investigating transient states, folding pathways, and functional conformational landscapes in complex biological systems.

## Materials and Methods

### Sample Preparation and dDNP Experiments

#### Dissolution DNP

For DNA experiments, 200 μL of a solution of 15 mM TEMPOL in a mixture of 30% glycerol-d_8_ and 70% H_2_O was flash frozen with liquid nitrogen and then inserted into a magnetic field of 6.7 T at a temperature of 1.4 K. Water was hyperpolarized by continuous-wave microwave irradiation at 188 GHz for 2 h. The magnet-cryostat combination was purchased from Cryogenic Ltd. and operated as described in reference.^45^ Solid-state polarization was detected using a 400 MHz Bruker NEO system adapted to a ^1^H resonance frequency of 285.3 MHz by using a broadband preamplifier.

The home-built detection circuit and the external tune-and-match system are described in references.^45,46^ The build-up was monitored by 1-degree flip-angle detection pulses applied every 5 s.

After DNP, the hyperpolarized water pellet was dissolved with a burst of superheated 5 mL D_2_O at 1.5 MPa as described in reference.^45^ The resulting concentration of the hyperpolarized H_2_O was 1.5% v/v. The hyperpolarized liquid was then pushed through a magnetic tunnel (*B*_tunnel_ = 0.5 T) with helium gas at 0.7 MPa to the hybrid sample shuttling system v2 (HySSS.v2)^37^ waiting in the detection spectrometer. The HySSS.v2 was connected to a Shigemi NMR tube prefilled with 150 μL of DNA solution. A home-built pressure heater actuated with an Arduino microcontroller was used for the dissolution process. The dissolution and injection steps were controlled using a home-written Python-based user interface. The entire process, from the dissolution of the DNP mixture to the start of NMR acquisition, took about 2.5 s (1.5 s transfer + 1 s settling delay).

Upon mixing the hyperpolarized water with the DNA (to a final sample volume of 600 μL), a 500 MHz Bruker NEO spectrometer equipped with a cryogenically cooled Prodigy BBO probe was used for detection. Detection was carried out as a series of single-pulse experiments using either selective excitation of imino and aromatic region (60-degree gauss1.1000 shaped pulse; for iTRP in the Supporting Information) or 1-degree hard pulse excitation across the whole spectral width. The excitation pulse was followed by 0.4 s acquisition time. The recycling delay was set to 1 s.

DNA samples were prepared at higher buffer concentrations to compensate for the 4-fold dilution by HyperW in the dDNP experiments.

T121-6: 500 μM T121-6-iM was prepared as in ref.^29^ DNA was dissolved in 50 mM potassium phosphate buffer with 100 mM KCl at pH = 6.3 in D_2_O. The sample was then incubated at 95 °C for 10 min. The dDNP experiments were then performed at 20 °C.

C9: 500 μM C9 iM was prepared as in ref.^47^ DNA was dissolved in 200 mM potassium phosphate with 400 mM KCl at pH = 6.9 in D_2_O. The sample was then incubated at 45 °C for 2 days to achieve stable folding. The dDNP experiments were then performed at 29.5 °C.

c-myc: 500 μM c-myc GQ was prepared as in ref.^30^ DNA was dissolved in 25 mM potassium phosphate buffer with 50 mM KCl at pH = 6 and annealed at 95 °C for 10 min. 150 μL of DNA was then pipetted into a 5 mm Shigemi NMR tube, inserted into the NMR magnet, and heated to 30 °C for dDNP experiment.

iTRP: 500 μM iTRP was prepared as in ref.^48^ The DNA sample was prepared in deuterated 30 mM potassium phosphate buffer, 300 mM KCl, 300 mM NaCl, 15 mM MgCl_2_ at pH 6. The sample was then annealed at 95 °C for 10 min dDNP was performed at 30 °C.

#### Glucose

DNP sample for glucose experiment was prepared by mixing 3 M glucose with 15 mM TEMPOL in 90% D_2_O and 10% H_2_O. Total volume of the mixture was 200 μL. dDNP measurement was done at 25 °C.

Dissolution DNP of glucose: dDNP of glucose was performed similarly to the DNA protocol, except that glucose was hyperpolarized directly with the TEMPOL radical. Upon dissolution, the sample was mixed with 150 μL of D_2_O (99.9%) in the NMR tube.

The signal enhancement ε was computed as the signal ratio of signal integrals *I* between the first detection in the time series and a reference recorded on the exact same sample after decay of the hyperpolarization, normalized by the number of scans *NS* and the detection flip angle θ:

$$\varepsilon =\frac{I_{hyperpolarized}{NS}_{reference}\sin\theta_{reference}}{I_{reference}{NS}_{hyperpolarized}\sin\theta_{hyperpolarized}}$$

However, it should be noted that the enhancement does not reflect the polarization of pure HyperW due to dilution with unpolarized protons upon dissolution and mixing. Indeed, the final sample contained a high concentration of water (protons), which contributes to the intensity of the water resonance in the reference spectrum. Correcting for this factor leads to a HyperW-representative ε of 420, in line with ε values reported under similar conditions.^24^

### Selective Suppression of Hyperpolarized Water Signal

Suppression of the dominant hyperpolarized water signal was achieved using a postprocessing approach based on low-rank Hankel matrix reconstruction (Cadzow denoising).^34,49^ This method exploits the strong temporal correlation of the water signal in the free induction decay (FID).

For each transient, the complex FID *x*(*n*), *n* = 1, ..., *N*, was truncated to an initial segment of length *N*_*s*_ (we used 1/4 of the total FID length), corresponding to a fraction of the total acquisition time:

$$x_{s}\left(n\right)=x\left(n\right),n=1, ...,N_{s}$$

From this truncated signal, a Hankel matrix $H\in C^{L\times \left(N_{s}-L+1\right)}$ was constructed as

$$H=\left[\begin{array}{cccc} x_{s}\left(1\right) & x_{s}\left(2\right) & \cdots & x_{s}\left(N_{s}-L+1\right)\\ x_{s}\left(2\right) & x_{s}\left(3\right) & \cdots & x_{s}\left(N_{s}-L+2\right)\\ \vdots & \vdots & \ddots & \vdots \\ x_{s}\left(L\right) & x_{s}\left(L+1\right) & \cdots & x_{s}\left(N_{s}\right) \end{array}\right]$$

with *L* being 1/3 of the total FID length. The Hankel matrix was decomposed using singular value decomposition (SVD):

$$H=U\Sigma V^{†}$$

where *U* and *V* are unitary matrices and Σ is a diagonal matrix containing singular values in decreasing order. The dominant water contribution was isolated by retaining only the first *r* singular values:

$$H_{r}=U_{r}\Sigma_{r}V_{r}^{†}$$

where *r* = *R*_water_ is the chosen rank. This truncation yields a low-rank approximation corresponding to the strongly correlated water signal.

The matrix *H*_*r*_ was iteratively (10x) projected back onto the space of Hankel matrices (Cadzow iterations) to enforce structural consistency. The resulting matrix was then converted back into a one-dimensional time-domain signal *x*_water_(*n*) by averaging along its antidiagonals:

$$x_{\mathrm{water}}\left(k\right)=\frac{1}{N_{k}}\underset{i+j=k+1}{\sum }{\left(H_{r}\right)}_{i,j}$$

where *N*_*k*_ is the number of elements along the corresponding antidiagonal.

The estimated water contribution was extended to the full FID and subtracted from the original signal:

$$x_{\mathrm{clean}}\left(n\right)=x\left(n\right)-x_{\mathrm{water}}\left(n\right)$$

The clean signal was subsequently transformed into the frequency domain via Fourier transformation, yielding the water-suppressed spectrum. The data was finally baseline-corrected using spline interpolation. The effectiveness of the method depends on the choice of truncation length *N*_*s*_ and rank *R*_water_. These parameters were optimized individually for each data set. In particular, *R*_water_ determines the degree of separation between the dominant water signal and weaker solute contributions. Because the characteristics of the water signal vary with sample composition and experimental conditions, optimal cutoff parameters must be adjusted on a per-sample basis to ensure efficient suppression without distorting solute resonances.

Finally, it should be noted that simple subtraction of a fitted NMR line did not lead to a satisfactory result, as the SVD-based approach (see Figure S8).

Full MATLAB codes for this operation are provided in the Supporting Information.

### Coupled Kinetic Modeling and Parameter Estimation

Time-resolved ^1^H NMR signals were analyzed using a coupled kinetic model describing polarization transfer and cross-relaxation across four magnetization reservoirs. The system was represented by a set of ordinary differential equations (ODEs) corresponding to the four magnetization reservoirs: hyperpolarized water (W), two signals of A12 and the 5′/3′ tail (I_1_ and I_2_), and the bulk contribution (E). The temporal evolution of the magnetization was modeled as

$$\frac{dW}{dt}=-\left(R_{W}+\sigma_{W1}+\sigma_{W2}\right)W$$

$$\frac{dI_{1}}{dt}=\sigma_{W1}W-R_{1}I_{1}$$

$$\frac{dI_{2}}{dt}=\sigma_{W2}W-R_{2}I_{2}$$

$$\frac{dE}{dt}=-R_{E}\left(E-E_{\infty }\right)$$

where *R*_*i*_ denote effective relaxation/decay rate constants, and σ_*W*1_, σ_*W*2_represent effective polarization transfer rates from hyperpolarized water to the loop/tail. Exchange terms between loops and the folded core were initially included but found to be negligible (σ = 0) and therefore omitted from the final reduced model (i.e., converged to negligible values and were omitted from the reduced equations above).

The system of ODEs was numerically integrated using a variable-step stiff solver (ode15s, MATLAB R2024b, MathWorks), appropriate for coupled kinetic systems with disparate time scales. Note that using a nonstiff solver (ode45) led to indistinguishable results.

Model parameters were estimated by simultaneous least-squares fitting of all experimentally observed time traces (W, I_1_, I_2_, and E). This ensured that a single, self-consistent parameter set described the full data set.

The mapping between simulated state variables and experimental observables was defined through linear scaling and offset parameters for each trace:

$$y_{j}\left(t\right)=s_{j}\cdot X_{j}\left(t\right)+b_{j}$$

where *X*_*j*_(*t*) is the signal intensity, and *s*_*j*_, *b*_*j*_ are fitted scaling and baseline parameters. These terms account for differences in detection sensitivity, normalization, and residual phase or baseline distortions, particularly relevant for signals approaching zero intensity.

Parameter estimation was performed using nonlinear least-squares minimization (lsqnonlin, trust-region-reflective algorithm). The objective function consisted of concatenated residuals across all traces:

$$\chi^{2}=\underset{j}{\sum }\underset{i}{\sum }w_{j}{\left[y_{j,\exp}\left(t_{i}\right)-y_{j,\mathrm{model}}\left(t_{i}\right)\right]}^{2}$$

where *w*_*j*_ are weighting factors chosen to normalize contributions from each trace and prevent dominance by signals with larger absolute amplitudes.

Physically meaningful bounds were imposed on all parameters during optimization (non-negative rate constants and constrained scaling factors). Multiple initial parameter guesses were tested to assess convergence behavior.

The optimization consistently converged to a stable solution; however, several parameters associated with exchange between loops and the folded core approached zero or their imposed bounds, indicating that these processes were not identifiable from the available data. Consequently, a reduced model excluding these exchange pathways was adopted for final analysis (vide supra).

Due to the limited number of time points and partial correlation between parameters, the extracted rate constants should be interpreted as effective kinetic parameters. The extracted transfer rates should not be interpreted as microscopic exchange rates between individual water molecules and specific imino/amino groups. Instead, they represent effective ensemble-averaged cross-relaxation parameters describing polarization transfer from the hyperpolarized water reservoir to the observed DNA magnetization.

The experimentally observed transfer rates incorporate the probability of water occupancy within the DNA hydration shell, the probability of water-DNA proton exchange, and the dipolar cross-relaxation efficiency. The fitted rates correspond to the cumulative efficiency of these processes.

Constant error bars of ±2 · 10^6^ a.u. were applied uniformly to all data points. During nonlinear least-squares fitting, however, each trace was weighted according to its variance to prevent high-amplitude signals from dominating the objective function. The weighting factors were defined as

$$w_{j}=\frac{1}{\sigma_{j}}$$

where σ_*i*_denotes the standard deviation of the normalized trace *i*. The objective function, therefore, minimized the weighted residual sum of squares across all four data sets simultaneously. Residuals were subsequently inspected as a function of time and showed no systematic drift, oscillatory structure, or temporal correlation, indicating that the reduced kinetic model adequately captured the dominant signal dynamics within experimental uncertainty. No evidence of systematic underfitting at early or late time points was observed.

## Supplementary Material



## Notes

DNA sequences, cutoff rank analysis, NOESY data, signal assignments, further examples on glucose and DNA, analysis of baseline distortions, and an example of a fit-and-subtract approach (PDF)
