# Supplementary material for: Unmasking DNA Resonances by Suppression of Hyperpolarized Water
Source: J Am Chem Soc. 2026 Jul 24;148(30):32297–306. doi: 10.1021/jacs.6c07731 (PMC13450397; doi:10.1021/jacs.6c07731)
Supplement: Supplementary file 1 [file ja6c07731_si_001.pdf]

Unmasking DNA Resonances by Suppression of Hyperpolarized Water

Milan Zachrdla[1], Ertan Turhan[1], Michala Bučková[2,3], Lukáš Trantírek[2], Dennis Kurzbach[1,]*

[1]Institute of Biological Chemistry, Faculty of Chemistry, University of Vienna, Währinger Str. 38, 1090 Vienna, Austria.

[2] Central European Institute of Technology, Masaryk University, 625 00, Brno, Czech Republic.

[3] National Centre for Biomolecular Research, Faculty of Science, Masaryk University, Kamenice 5, 625 00 Brno, Czech Republic

**dennis.kurzbach@univie.ac.at*

Supporting Information

Table S1. List of DNA constructs employed in the present study.

Name	Structural motif	Sequence (5' → 3')	Ref.
c-myc	G-quadruplex	TGAGGGTGGGTAGGGTGGGTAA	[1]
T121-6	i-motif	CCCCCCTCCCCCCTTCCCCCCTCCCCCC	[2]
iTRP	Triplex/H-DNA	GAGAGAACCCCTTCTCTCTTTCTCTCTT	[3]
C9	i-motif	$(CCCCCCCCCTTT)_3$CCCCCCCCC	[4]

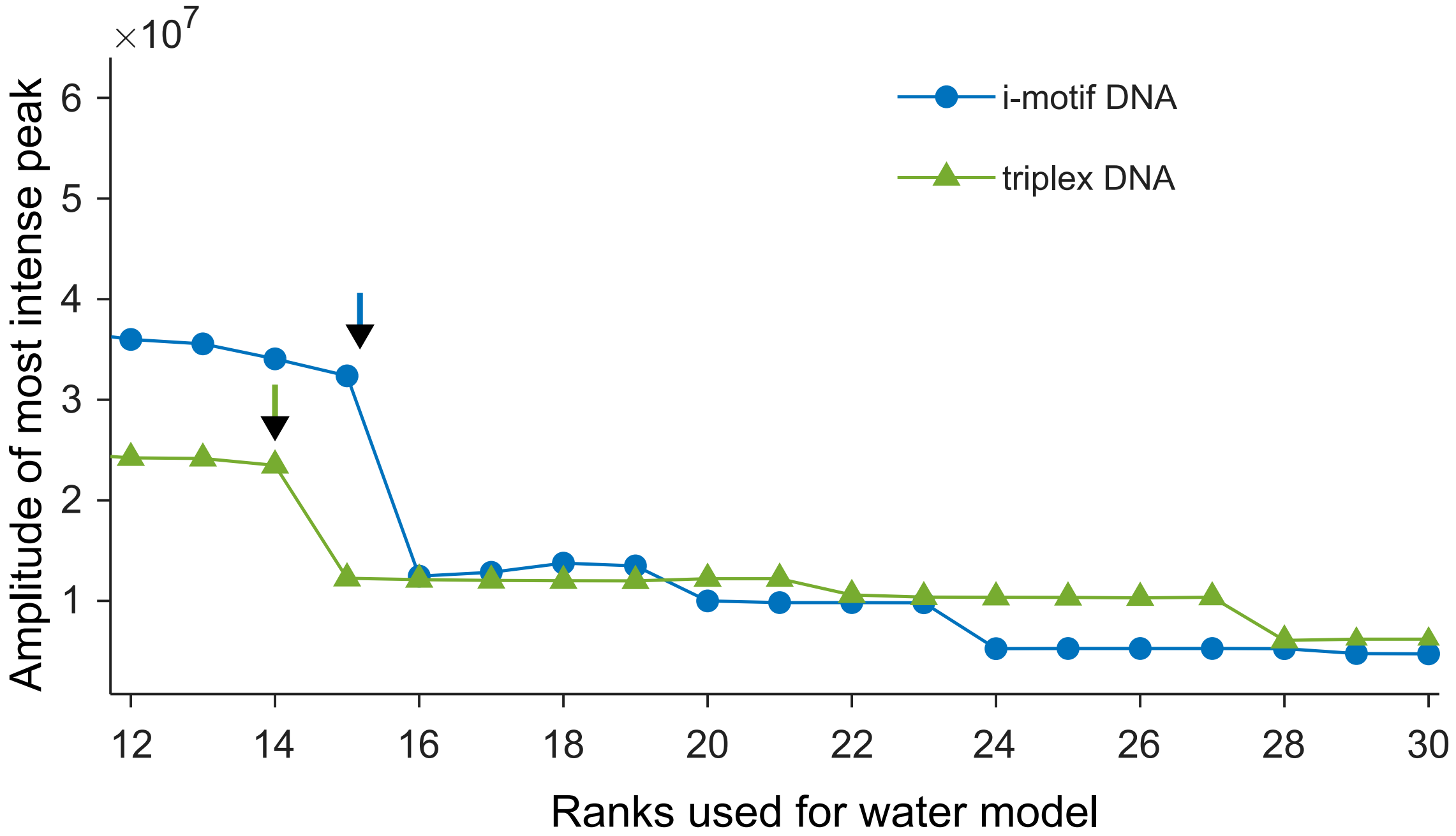

Figure S1. Dependence of analyte signal intensity on the number of singular values retained during reconstruction of the HyperW contribution. The figure shows the intensity of selected DNA resonances as a function of the SVD rank used for water reconstruction. At low ranks, the dominant water signal is selectively captured and removed, while the analyte signals remain unaffected. As the rank increases beyond a threshold (indicated by the arrow), the singular values begin to include contributions from the analyte, leading to a decrease in signal intensity. This behavior demonstrates that efficient separation relies on a clear amplitude disparity between the water and analyte signals and defines an optimal rank range for preserving spectral integrity.

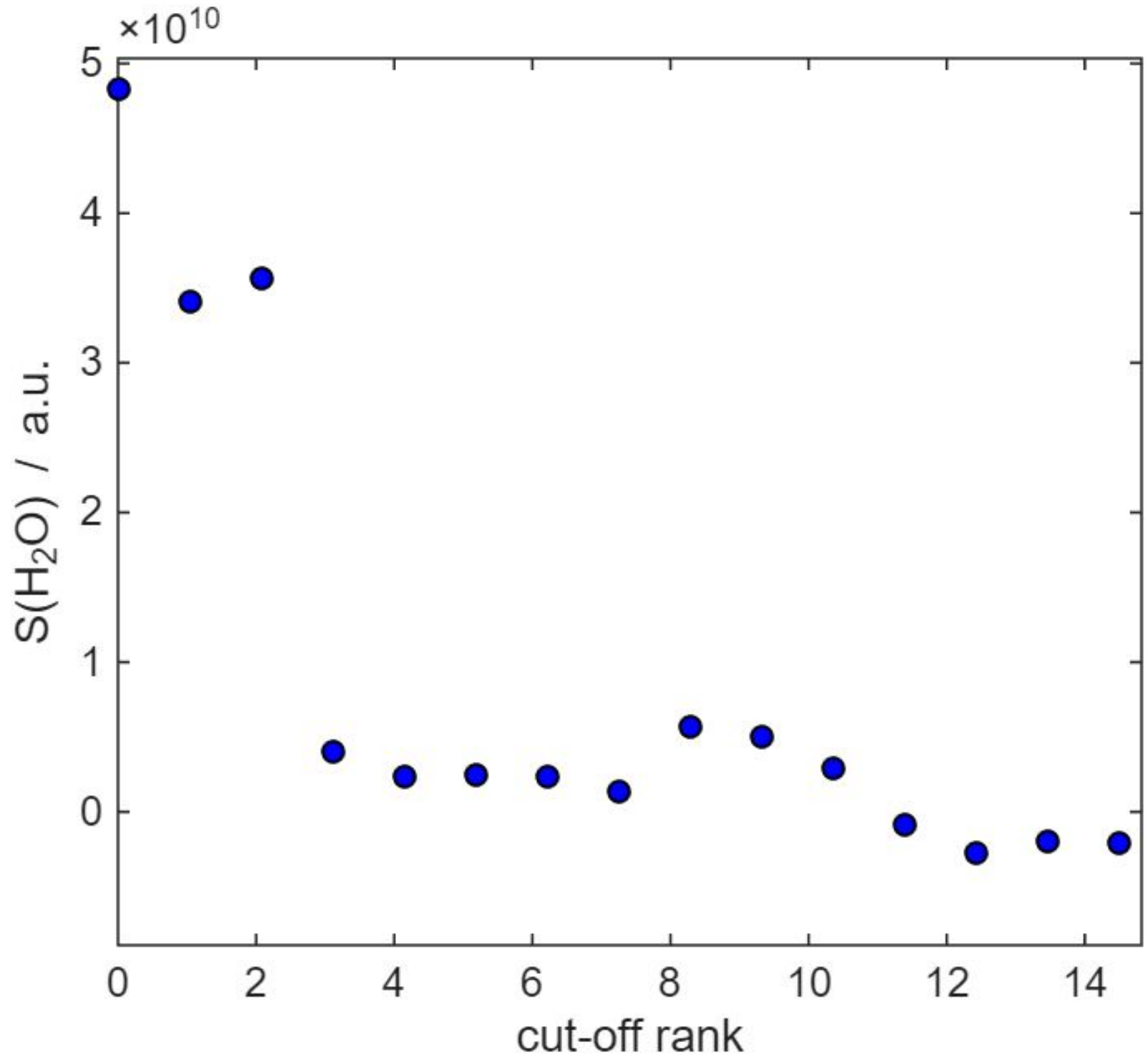

Figure S2. Residual water signal intensity as a function of SVD cut-off rank. The plot shows the remaining water signal amplitude after subtraction as a function of the number of singular values used in reconstruction. A rapid decrease in water intensity is observed with increasing rank, reaching near-complete suppression at approximately 12 singular values. In combination with Fig. S1, this indicates that HyperW can be suppressed when using 12 as a cut-off rank (i.e. 12 singular values for reconstruction), while the analyte signals remain stable until a rank of 14. These results define an optimal parameter window in which water suppression is maximized while preserving biomolecular signal fidelity. Note that for other systems the optimal cut-off might be different.

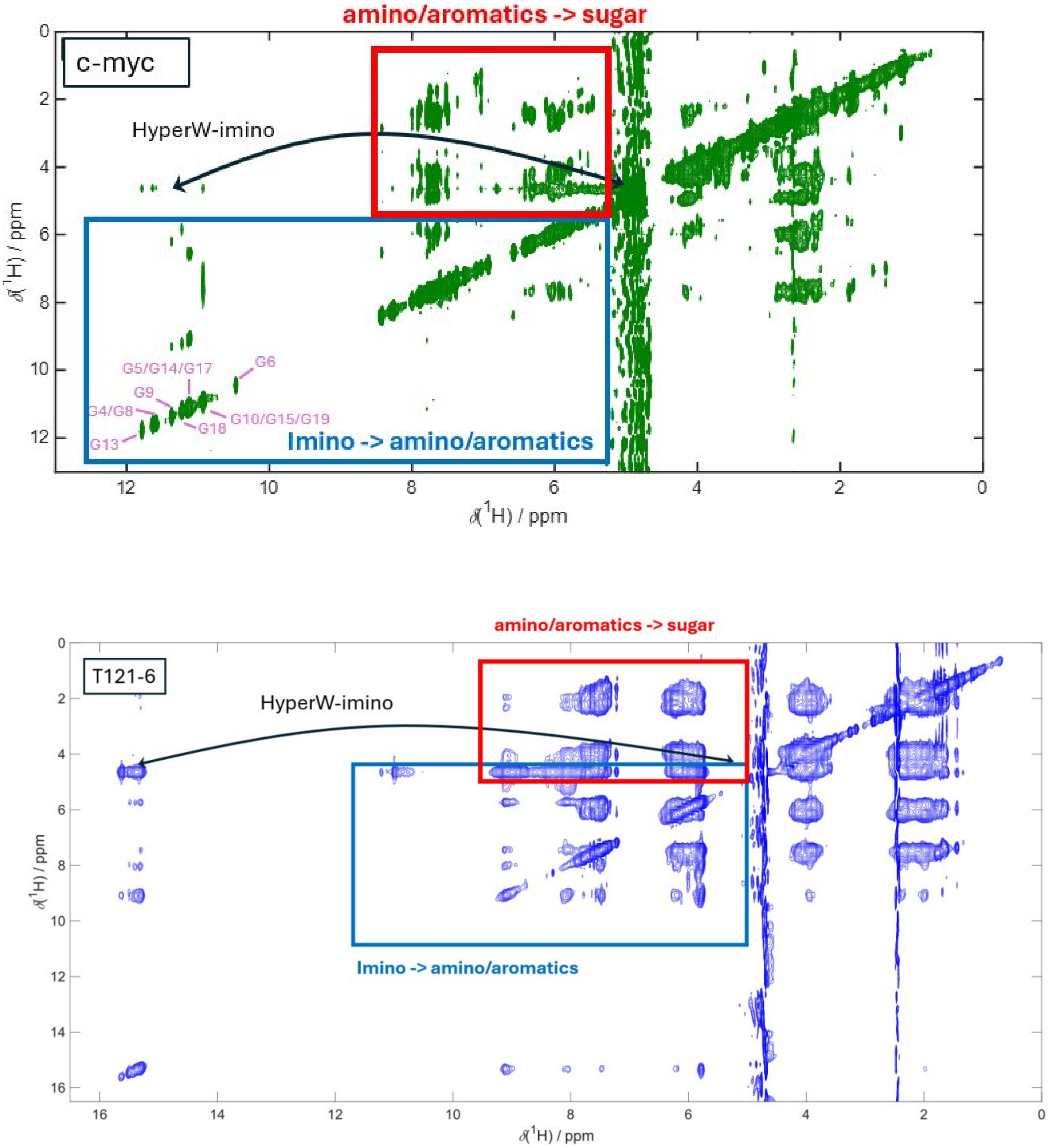

Figure S3. c-myc and T121-6 NOESY highlighting magnetization transfer pathways relevant for HyperW-mediated polarization relay. The spectrum displays cross-peaks between exchangeable imino/amino protons and aromatic resonances, as well as subsequent correlations to deoxyribose protons. These connectivity patterns illustrate a sequential polarization transfer pathway: initial exchange of HyperW with labile imino/amino protons, followed by NOE-mediated transfer to aromatic sites, and subsequent propagation to the sugar backbone. The observed NOE network supports the interpretation of the site-specific hyperpolarization patterns observed in the dDNP experiments.

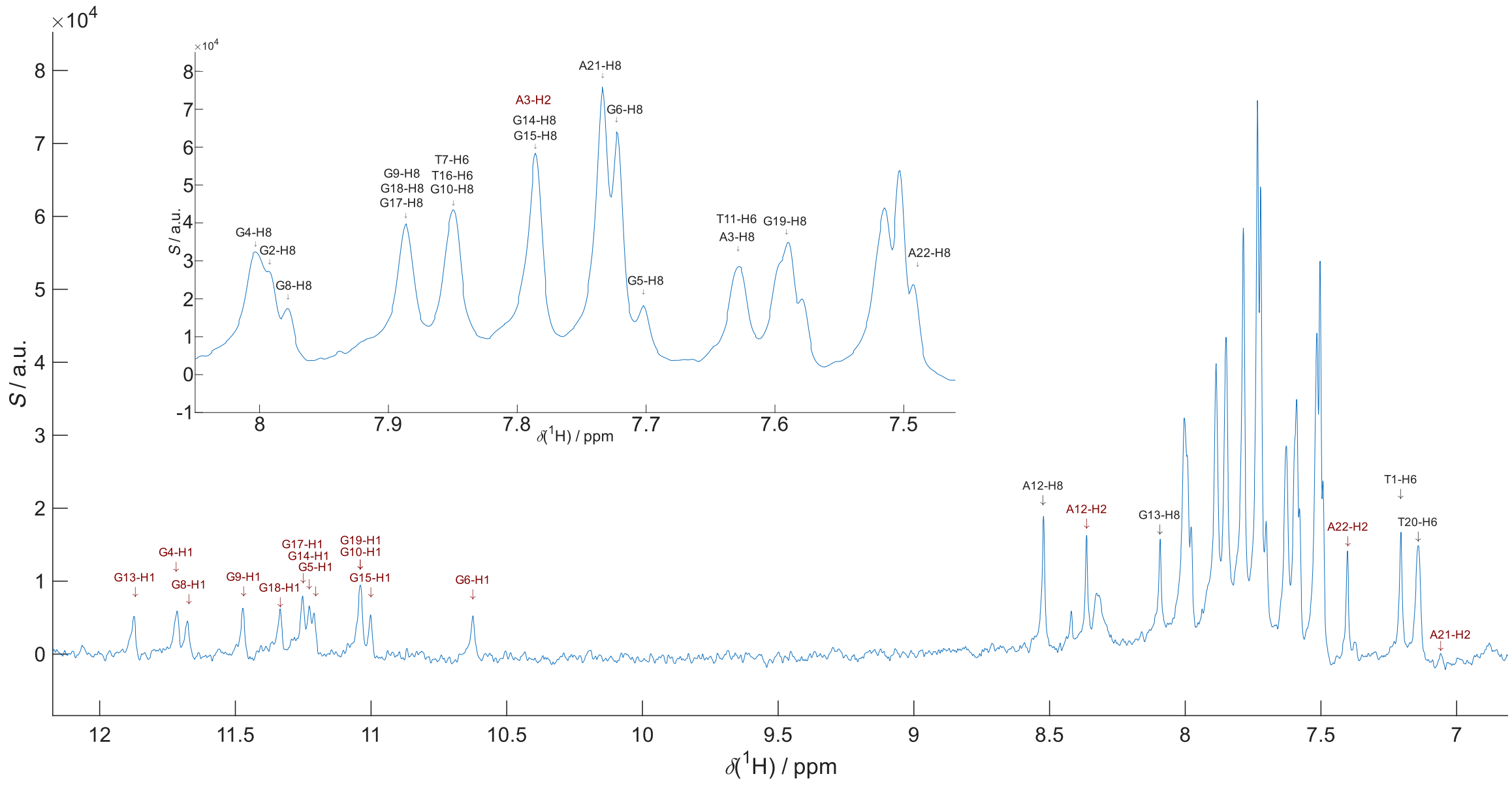

Figure S4. Signal assignment for c-myc.[1]

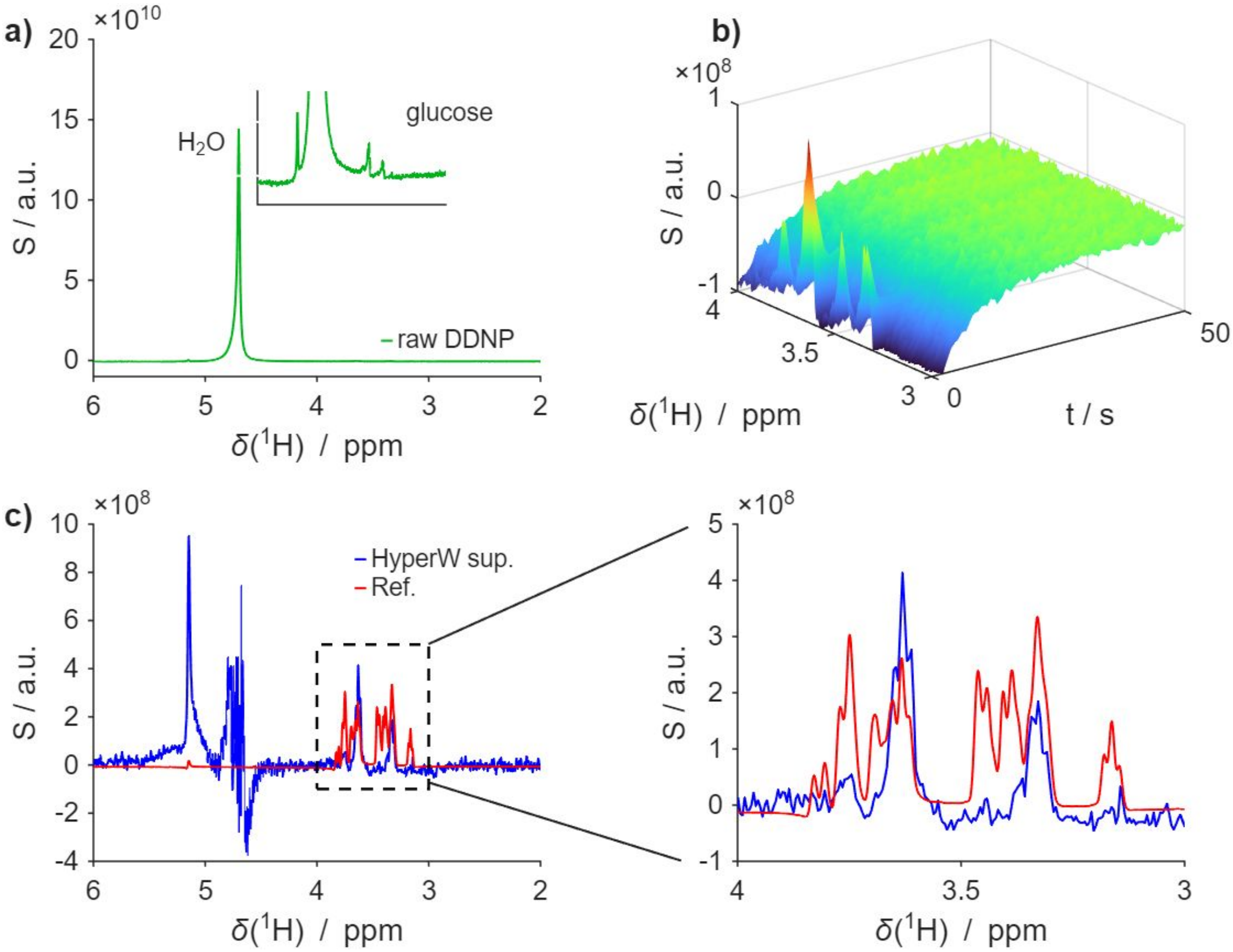

Figure S5. Additional example 1: Application of the HyperW suppression procedure to a small-molecule system highlights the generality of the approach beyond nucleic acids. In this experiment, glucose was directly hyperpolarized during the dDNP process (i.e., polarization originates from the DNP step, not from HyperW-mediated transfer), providing a complementary test case with distinct polarization pathways.

a) Raw hyperpolarized ^{1}H spectrum recorded immediately after injection, dominated by a broad and intense water signal accompanied by strong baseline distortions, which obscure most glucose resonances.

b) Time evolution of the hyperpolarized spectrum (1° flip angle, 1 s repetition), showing the simultaneous decay of glucose signals and the HyperW background. The spectral region remains largely inaccessible due to the dominant solvent contribution and associated distortions throughout the detection period.

c) Spectrum after SVD-based removal of the water contribution, revealing well-resolved glucose resonances across the full spectral range. Chemical shifts and linewidths are consistent with the conventional reference spectrum (sum of 128 scans, 90° flip angle), confirming that the subtraction procedure preserves spectral fidelity.

Variations in signal intensities across resonances reflect differences in direct hyperpolarization efficiency and subsequent polarization transfer pathways within the molecule. Note the strong enhancement of the anomeric signal at >5 ppm.

These data demonstrate that the method is not restricted to nucleic-acid systems or HyperW-mediated polarization transfer, but is broadly applicable to directly hyperpolarized small molecules, providing reliable spectral recovery under dDNP conditions.

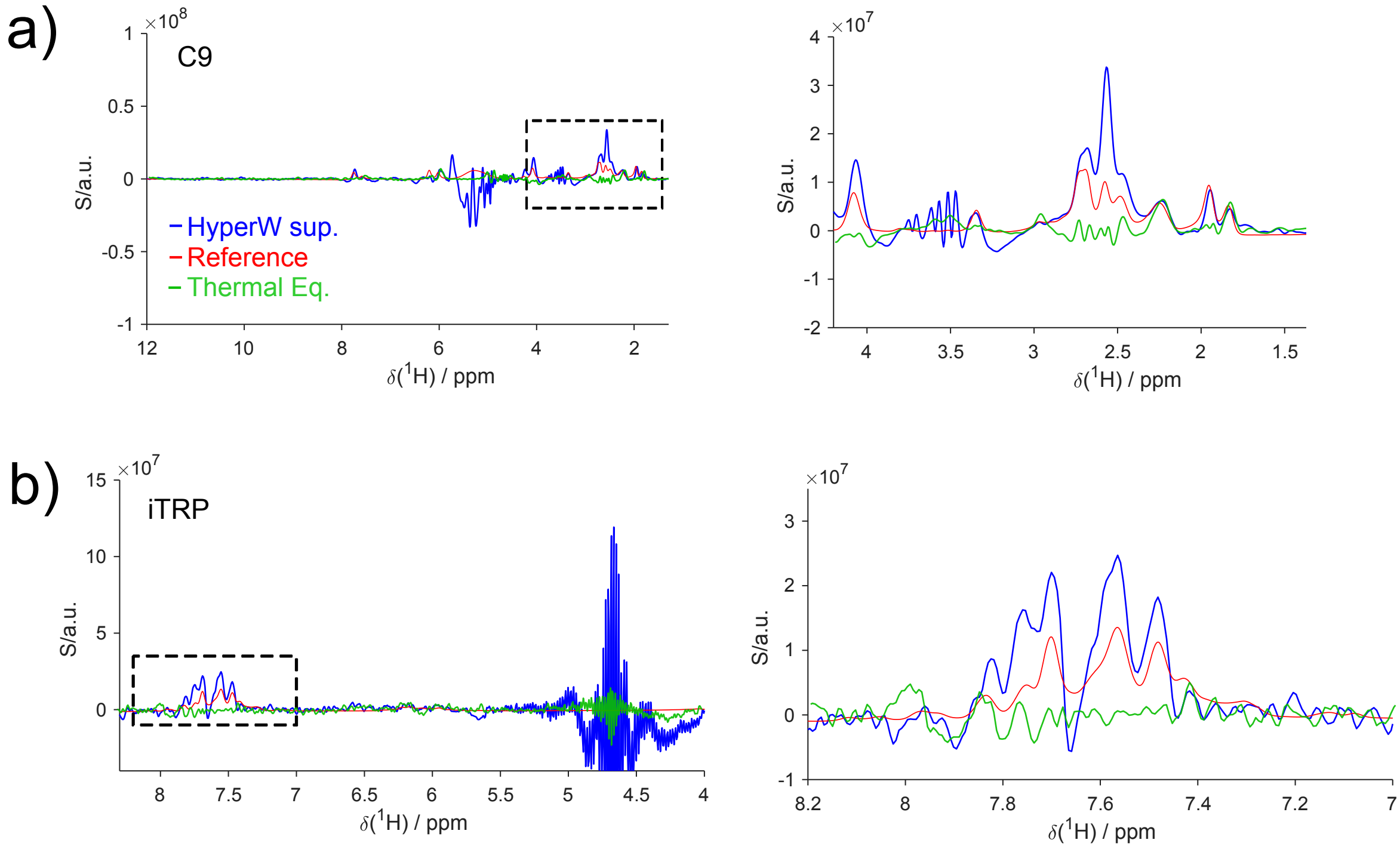

Figure S6. Additional examples 2 and 3: Application of HyperW suppression to additional DNA constructs, demonstrating robustness across different nucleic-acid systems.

a) C9 DNA. Comparison of the HyperW-suppressed hyperpolarized spectrum (blue; 3 scans; 1° flip angle) with a spectrum recorded after complete decay of hyperpolarization (thermal reference, green; 1 scan; 1° flip angle) demonstrates accurate recovery of resonance positions and linewidths across the full spectral range. A high-sensitivity conventional reference spectrum (grey; 256 scans; 90° flip angle) is shown for comparison. The suppressed spectrum reproduces the expected chemical shifts while exhibiting substantial signal enhancement, confirming that the subtraction procedure does not distort the underlying spectral information.

b) iTRP DNA. Analogous comparison for a triplex/H-DNA construct measured under identical HyperW conditions (blue; 3 scans; 1° flip angle), together with the corresponding thermal reference (green; 1 scan; 1° flip angle) and conventional reference spectrum (grey; 256 scans; 90° flip angle). As in panel (a), the HyperW-suppressed spectrum faithfully reproduces resonance positions and linewidths while revealing heterogeneous hyperpolarization across different sites.

In both systems, the HyperW-suppressed spectra provide broadband spectral access that is otherwise obscured in the raw hyperpolarized data. The agreement with conventional reference spectra confirms that the procedure preserves spectral fidelity, while the enhanced signal intensities reflect efficient transfer of hyperpolarization.

These results demonstrate that the approach is robust across different nucleic-acid topologies and is not limited to a specific DNA motif.

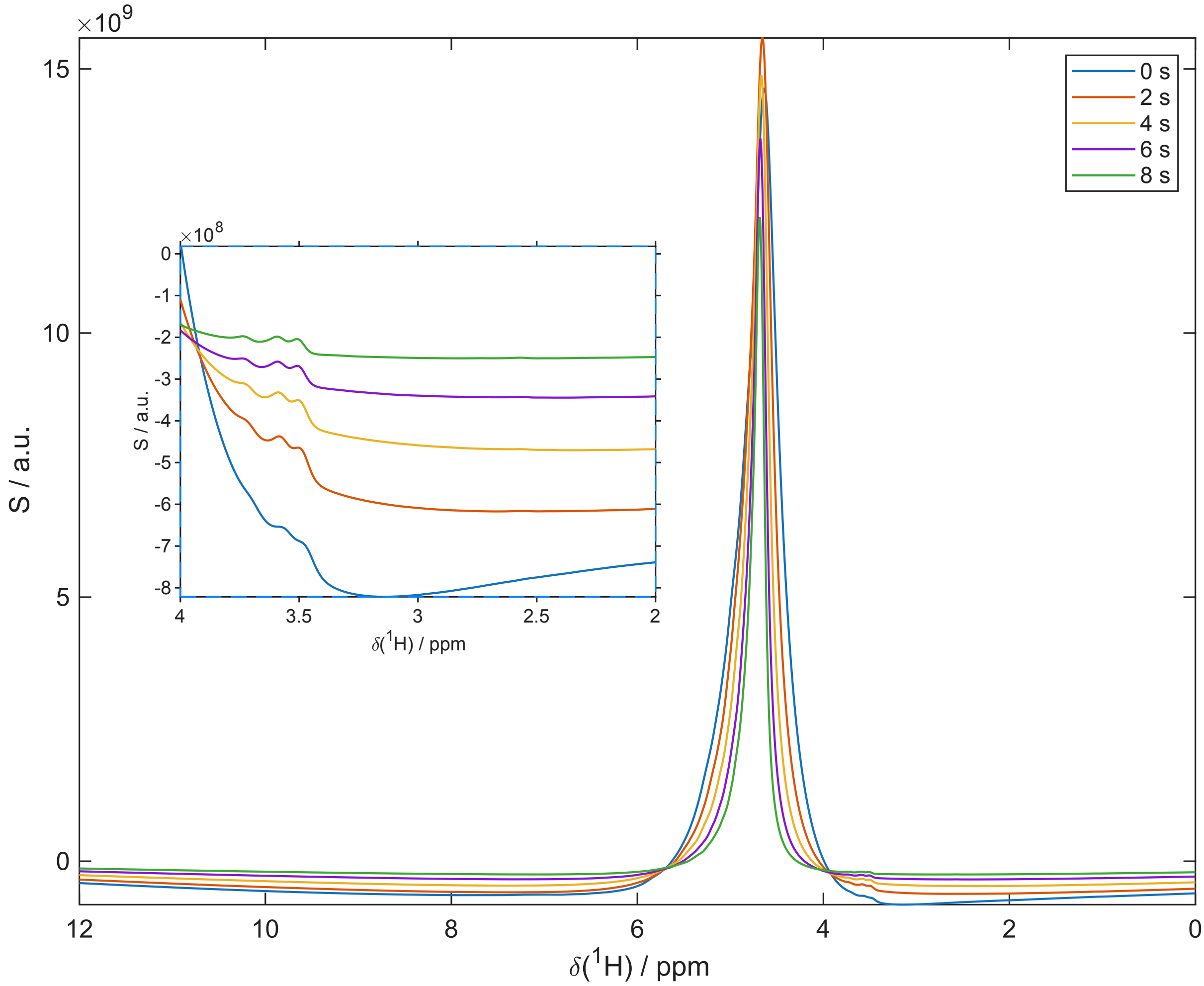

Figure S7. Non-corrected time-series of ^{1}H NMR spectra after injection of HyperW and mixing with the target DNA solution. The legend indicates the time elapsed since completion of the mixing process. With time, the water resonance at 4.7 ppm decays. Importantly, the baseline distortions are correlated with the HyperW signal intensity. As the hyperpolarization decays, distortions diminish as well (see inset).

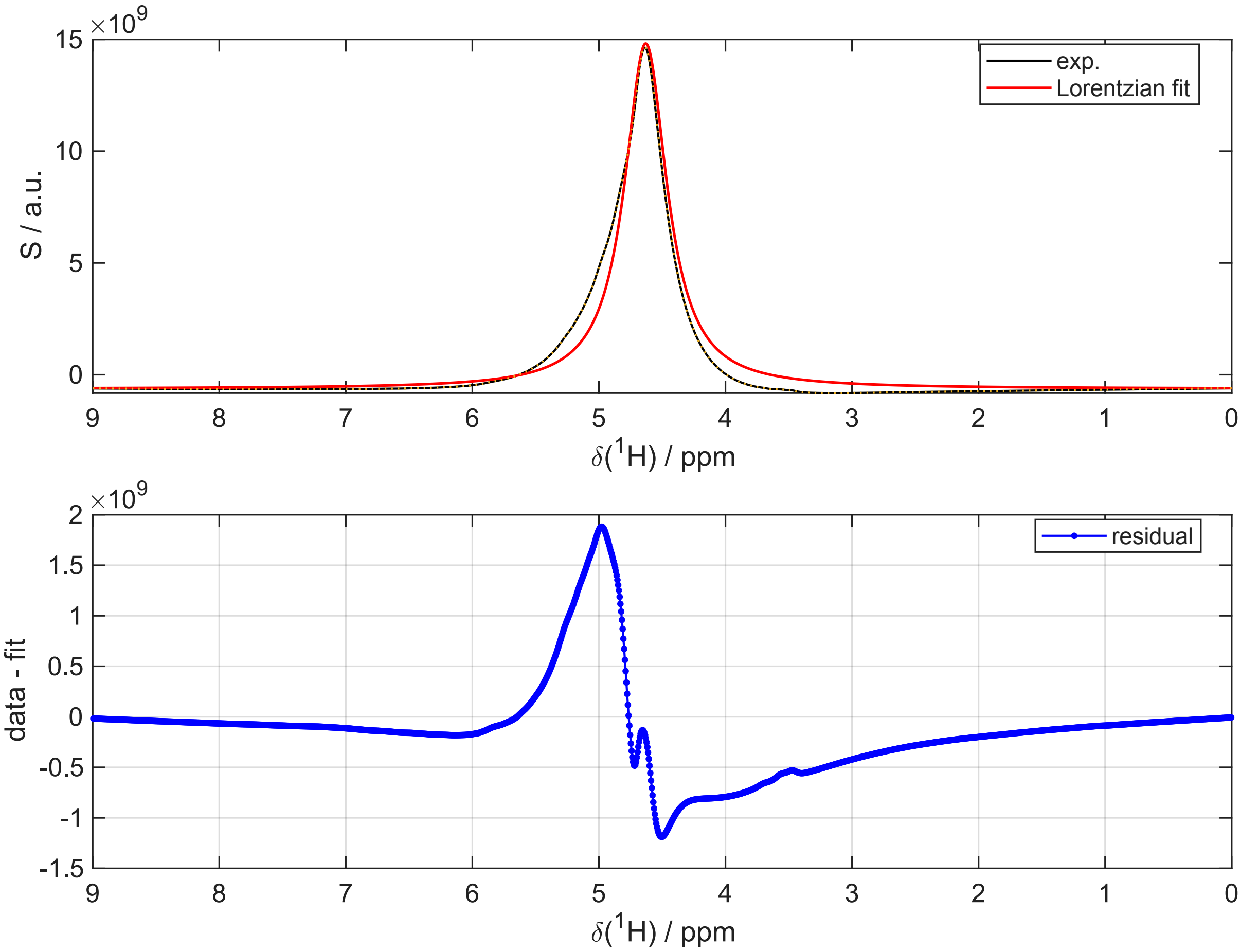

Figure S8. Top: Raw HyperW signal detected after mixing with the DNA solution (black) and a Lorentzian fit (red). Bottom: Subtraction of experimental data and the fit. The residual after subtraction is shown in blue. Removing the HyperW contribution via fitting and subtraction does not yield a clean baseline, unlike the SVD approach shown in the main text. The hyperpolarized water signal deviates too much from ideal line shapes, primarily due to radiation damping, so it cannot be fitted with a Lorentzian line shape.

MATLAB Functions for HyperW subtraction

```
%% Functions
function out = removeWaterCadzow(fid, swHz, opts)
%REMOVEWATERCADZOW Remove dominant broad water using Cadzow-style Hankel SVD
%
% Example:
%   out = removeWaterCadzow(fid, swHz, RWater=2, ShortFrac=0.2);

arguments
    fid (:,:) double
    swHz (1,1) double

    opts.HankelRows (1,1) double = NaN
    opts.ShortFrac (1,1) double = 0.25
    opts.RWater (1,1) double = 2
    opts.NumIter (1,1) double = 2
    opts.PlotResult (1,1) logical = false
end

x = fid(:);
N = numel(x);
dt = 1/swHz;
t = (0:N-1).' * dt;

if abs(x(1)) > 0
    x = x .* exp(-1i*angle(x(1)));
end

Ns = max(64, min(N, round(opts.ShortFrac * N)));
xs = x(1:Ns);

L = opts.HankelRows;
if isnan(L)
    L = round(Ns/2);
end
L = max(8, min(L, Ns-1));

% Hankel matrix
H = hankel(xs(1:L), xs(L:Ns));

% Cadzow iterations
for it = 1:opts.NumIter
    [U,S,V] = svd(H, "econ");
    r = min(opts.RWater, min(size(S)));
    Hlow = U(:,1:r) * S(1:r,1:r) * V(:,1:r)';
    H = enforceHankel(Hlow);
end

% Convert Hankel water estimate back to 1D
xWaterShort = antiDiagAverage(H);

% Put into full-length FID
xWater = zeros(N,1);
xWater(1:Ns) = xWaterShort;

% Smooth taper at the end of fitted segment
nTaper = min(32, floor(Ns/4));
if nTaper >= 4
```

```
    w = ones(Ns,1);
    taper = 0.5 * (1 + cos(pi*(0:nTaper-1)'/(nTaper-1)));
    w(end-nTaper+1:end) = taper;
    xWater(1:Ns) = xWater(1:Ns) .* w;
end

xClean = x - xWater;

Xraw   = fftshift(fft(x));
Xwater = fftshift(fft(xWater));
Xclean = fftshift(fft(xClean));
fHz    = linspace(-swHz/2, swHz/2, N).';

out = struct;
out.fidRaw     = x;
out.fidWater   = xWater;
out.fidClean   = xClean;
out.specRaw    = Xraw;
out.specWater  = Xwater;
out.specClean  = Xclean;
out.fHz        = fHz;
out.time       = t;

if opts.PlotResult
    figure("Color","w");
    tiledlayout(3,1)

    nexttile
    plot(fHz, real(Xraw), "Color",[0.7 0.7 0.7], "LineWidth",1); hold on
    plot(fHz, real(Xwater), "b-", "LineWidth",1.2)
    plot(fHz, real(Xclean), "k-", "LineWidth",1.1)
    xlabel("Hz")
    ylabel("Real spectrum")
    legend("Raw","Estimated water","Clean","Location","best")
    title(sprintf("Cadzow water removal, RWater = %d", opts.RWater))
    grid on

    nexttile
    plot(t, real(x), "Color",[0.7 0.7 0.7], "LineWidth",1); hold on
    plot(t, real(xWater), "b-", "LineWidth",1.2)
    plot(t, real(xClean), "k-", "LineWidth",1.1)
    xlabel("s")
    ylabel("Real FID")
    legend("Raw","Estimated water","Clean","Location","best")
    grid on

    nexttile
    [~,Sshow,~] = svd(hankel(xs(1:L), xs(L:Ns)), "econ");
    stem(diag(Sshow), "filled")
    xlabel("Index")
    ylabel("Singular value")
    title("Early-FID Hankel singular values")
    grid on
end
end

function Hh = enforceHankel(H)
[m,n] = size(H);
Hh = zeros(m,n,class(H));
```

```
for s = 1:(m+n-1)
    ii = max(1, s-n+1):min(m, s);
    vals = zeros(numel(ii),1,class(H));
    for k = 1:numel(ii)
        i = ii(k);
        j = s - i + 1;
        vals(k) = H(i,j);
    end
    a = mean(vals);
    for k = 1:numel(ii)
        i = ii(k);
        j = s - i + 1;
        Hh(i,j) = a;
    end
end
end

function x = antiDiagAverage(H)
[m,n] = size(H);
x = zeros(m+n-1,1,class(H));
for s = 1:(m+n-1)
    ii = max(1, s-n+1):min(m, s);
    vals = zeros(numel(ii),1,class(H));
    for k = 1:numel(ii)
        i = ii(k);
        j = s - i + 1;
        vals(k) = H(i,j);
    end
    x(s) = mean(vals);
end
end
```

References

1. Ambrus, A.; Chen, D.; Dai, J.; Jones, R. A.; Yang, D., Solution structure of the biologically relevant G-quadruplex element in the human c-MYC promoter. Implications for G-quadruplex stabilization, *Biochemistry* **2005,** *44* (6), 2048-58. 10.1021/bi048242p

2. Cheng, M.; Qiu, D.; Tamon, L.; Istvankova, E.; Viskova, P.; Amrane, S.; Guedin, A.; Chen, J.; Lacroix, L.; Ju, H.; Trantirek, L.; Sahakyan, A. B.; Zhou, J.; Mergny, J. L., Thermal and pH Stabilities of i-DNA: Confronting in vitro Experiments with Models and In-Cell NMR Data, *Angew Chem Int Ed Engl* **2021,** *60* (18), 10286-10294. 10.1002/anie.202016801

3. Sklenar, V.; Feigon, J., Formation of a stable triplex from a single DNA strand, *Nature* **1990,** *345* (6278), 836-8. 10.1038/345836a0

4. Školáková, P.; Gajarský, M.; Palacký, J.; Šubert, D.; Renčiuk, D.; Trantírek, L.; Mergny,
J.-L.; Vorlíčková, M., DNA i-motif formation at neutral pH is driven by kinetic partitioning. *Nucleic Acids Res*. **2023**, 51 (6), 2950-2962. doi: 10.1093/nar/gkad119
